# Identifying Daily Living Skills From Childhood and Adolescence Predictive of Adult Outcomes in a Longitudinal Study of Autism and Related Developmental Conditions

**DOI:** 10.1002/aur.70056

**Published:** 2025-05-30

**Authors:** Elaine B. Clarke, Catherine Lord

**Affiliations:** ^1^ Rutgers, The State University of New Jersey, Graduate School of Applied and Professional Psychology Piscataway New Jersey USA; ^2^ Department of Psychiatry and Biobehavioral Sciences University of California, Los Angeles Los Angeles California USA

## Abstract

Challenges in daily living skills (DLS) are well‐documented in autism and other developmental conditions. Research has also cataloged challenges in adult outcome attainment among autistic individuals and those with other developmental conditions; stronger DLS are associated with a higher likelihood of attaining some adult outcomes. Little work has examined whether competency in *specific* DLS increases the likelihood of attaining adult outcomes. The current study examined mean item set scores from the DLS domain of the first and second editions of the Vineland Adaptive Behavior Scales (VABS) in a sample (*n* = 230) drawn from a well‐characterized longitudinal cohort. Differences in growth patterns in DLS item set scores based on cognitive ability were examined from ages 5–18. The utility of DLS item set scores from ages 5, 9, 14, and 18 for predicting adult employment, relationship, living, and well‐being outcomes at approximately age 33 was then tested. For all participants, DLS item sets from the community subdomain (i.e., eating out skills, pre‐job skills) were low throughout childhood and showed the least growth over time. For participants with IQ < 70, personal subdomain item sets (i.e., bathing, health) had the most predictive utility. For participants with IQ > 70, community subdomain item sets had the most predictive utility. Competency in personal DLS may promote positive outcomes for autistic individuals with IQ < 70; competency in community DLS may be more important to supporting outcomes for autistic individuals with average or higher IQ. These results could inform interventions intended to promote adult success.

**Summary**

- This study looked at daily living skills (DLS) in autistic individuals from childhood to adulthood and how these skills relate to adult success.
- DLS scores from the Vineland Adaptive Behavior Scales of 230 individuals were analyzed, examining both growth patterns and predictive value of specific skills.
- Personal skills (like bathing and health) were most useful for predicting positive outcomes for individuals with lower IQ, while community skills (like eating out and job skills) were more predictive for those with average or higher IQ.
- These findings can guide targeted interventions to support outcomes for autistic individuals.

Success in adult life is contingent upon completing small but meaningful daily tasks. We must get dressed, do laundry, and pay our bills—if we neglect these things, the consequences can be considerable. The completion of age‐salient tasks necessary for personal independence can be defined as daily living skills (DLS)—one domain of the larger construct of adaptive behavior (Sparrow and Cicchetti 1985). DLS can be further divided into personal (e.g., toileting, bathing, and grooming), domestic (e.g., cooking, household chores), and community (e.g., obeying traffic signals, using money, ordering in a restaurant; Table 1).

**TABLE 1:** DLS item sets comprised of overlapping items from the first and second editions of the VABS.

DLS subdomain	Item set	Items
Personal	Eating and drinking	Opens mouth when food is offered. Eats solid foods (e.g., cooked vegetables, chopped meats, etc.). Sucks or chews on finger foods (e.g., crackers, cookies, toast, etc.). Drinks from a cup or glass; may spill. Feeds self with spoon; may spill. Sucks from straw. Feeds self with fork; may spill. Feeds self with spoon without spilling. Holds spoon, fork, and knife correctly.
	Toileting	Let's someone know when he or she has wet or soiled diaper or pants. Urinates in toilet or potty chair. Asks to use toilet. Defecates in toilet or potty chair. Is toilet‐trained during the night.
	Dressing	Takes off clothing that opens in the front (e.g., a coat or sweater); does not have to unbutton or unzip the clothing. Pulls up clothing with elastic waistbands (e.g., underwear or sweatpants). Puts shoes on correct feet; does not need to tie laces. Wears appropriate clothing during wet or cold weather (e.g., raincoat, boots, sweater, etc.).
	Bathing	Washes and dries face using soap and water. Bathes or showers and dries self.
	Grooming	Brushes teeth. Washes and dries hair (with towel or hair dryer).
	Health	Wipes or blows nose using tissue or handkerchief. Covers mouth and nose when coughing and sneezing.
Domestic	Safety at home	Is careful around hot objects (e.g., the stove or oven, an open fire, etc.).
	Kitchen chores	Clears breakable items from own place at table. Helps prepare foods that require mixing and cooking (e.g., cake or cookie mixes, macaroni and cheese, etc.). Uses stove or oven for heating, baking, or cooking (i.e., turns burners on and off, sets oven temperature, etc.). Prepares food from ingredients that require measuring, mixing, and cooking. Plans and prepares main meal of the day.
	Housekeeping	Helps with simple household chores (e.g., dusts, picks up clothes or toys, feeds pet, etc.). Puts away personal possessions (e.g., toys, books, magazines, etc.). Puts clean clothes away in proper place (e.g., in drawers or closet, on hooks, etc.). Uses tools (e.g., a hammer to drive nails, a screwdriver to screw and unscrew screws, etc.). Sweeps mops or vacuums floors thoroughly. Uses household products correctly (e.g., laundry detergent, furniture polish, glass cleaner, etc.). Cleans one or more rooms other than own bedroom. Washes clothing as needed. Performs maintenance tasks as needed (e.g., replaces light bulbs, changes vacuum cleaner bag, etc.).
Community	Telephone skills	Talks to familiar person on telephone. Summons to the telephone the person receiving a call or indicates that the person is not available. Makes telephone calls to others, using standard or cell phone.
	Rules, rights, and safety	Is aware of and demonstrates appropriate behavior while riding in car (e.g., keeps seat belt on, refrains from distracting driver, etc.). Looks both ways when crossing streets or roads. Obeys traffic lights and “Walk” and “Don't Walk” signs. Demonstrates knowledge of what phone number to call in an emergency when asked.
	Time and dates	Demonstrates understanding of function of clock (e.g., says, “Clocks tell time”; “What time can we go?”; etc.). Says current day of the week when asked. Points to current or other date on calendar when asked. Tells time by 5‐min segments on analog clock (e.g., 1:05, 1:10, etc.).
	Money skills	Demonstrates understanding of the function of money (e.g., says, “Money is what you need to buy things at the store”; etc.). States value of penny (1 cent), nickel (5 cents), dime (10 cents), and quarter (25 cents). Counts change from a purchase. Uses savings or checking account responsibly (e.g., keeps some money in account, tracks balance carefully, etc.). Manages own money (e.g., pays most or all own expenses, uses checks or money orders for purchases as needed, etc.). Budgets for monthly expenses (e.g., utilities, rent, etc.).
	Eating out skills	Orders a complete meal in a fast‐food restaurant.
	Pre‐job skills	Obeys time limits for breaks (for example, lunch or coffee breaks, etc.). Notifies school or supervisor when he or she will be late or absent. Holds full‐time job responsibly.

Nearly 50 years of research has documented DLS challenges in autism^1^ as well as other developmental conditions. Notably, deficits in DLS and other adaptive behaviors are a core diagnostic criterion for intellectual disability (ID; American Psychiatric Association 2022). In autism, DLS difficulties emerge early in development (Bradshaw et al. 2019) and appear to persist into adulthood (Clarke, McCauley, et al. 2021; Teh et al. 2024). Poor DLS is linked to a lower likelihood of achieving milestones associated with adult life in the general population, such as attending post‐secondary education (Clarke, McCauley, et al. 2021), employment (Taylor et al. 2014), and living independently (Dudley et al. 2019). This appears to be true for both autistic adults and adults with other developmental conditions (Lord et al. 2020). However, given the array of behaviors that comprise daily living skills, it is unclear whether certain DLS are more important to attaining adult outcomes than others. Few studies have examined associations between specific DLS and specific adult outcomes, though such investigations could be critical to informing interventions to support greater independence (Glover et al. 2023; Teh et al. 2024).

## 1 DLS Development

As a developmental condition, autism both changes and is changed by development. In other words, core features of autism can change the way individuals interact with and learn about their environment from the earliest days of life, thus altering developmental trajectories (Bradshaw et al. 2022). Simultaneously, core features of autism are not static but can change markedly within a single individual with increasing age (Masjedi et al. 2024). Childhood acquisition of DLS provides fundamental skills necessary for later acquisition of more complex DLS (Chen et al. 2024; Di Rezze et al. 2019). It would be difficult to learn to cook scrambled eggs if you did not already know how to safely use a stove, for example. The current study examines how DLS from mid‐childhood through adolescence relates to adult outcomes.

Several studies have examined DLS domain and subdomain (i.e., personal, domestic, community; Table 1) scores in autism (Bal et al. 2015; Chen et al. 2024). Given the variability in individuals' profiles of strengths and challenges, summary scores can indicate the overall level of impairment in DLS but have limited utility for individualized support planning. DLS can be further divided into item sets—groups of several items related to the same type of life skill (Table 1). Little research to date has examined DLS at the level of item sets (Teh et al. 2024).

## 2 Defining Adult Success

A meta‐analysis of 18 adult outcome studies by Mason and colleagues (2021) found only 20% of autistic adults had attained “good” outcomes—defined as living independently, having a paid job, and having meaningful social relationships. These challenges persist even as academic achievement (Kim et al. 2017) and features of autism improve in adulthood (Bal et al. 2018; Seltzer et al. 2003, 2004). Research suggests that these challenges are not unique to autistic adults; adults with other developmental conditions also experience challenges attaining success in adulthood (Biggs and Carter 2016; DaWalt et al. 2019). Given the DLS challenges characteristic of many developmental conditions, perhaps these findings are unsurprising. Without competent daily living skills, it may be difficult to achieve many milestones associated with adult life in the general population, including full‐time employment and living independently (Duncan et al. 2018; McCauley, Elias, et al. 2020).

Adult outcome studies have recently been criticized for emphasizing indicators of success that may not be meaningful for all autistic adults (Orsmond et al. 2024; Pukki et al. 2022). Moreover, for individuals with autism and co‐occurring intellectual disabilities (ID), who are estimated to represent one‐third of people with autism (Zeidan et al. 2022), living independently and finding paid employment may not be attainable adult outcomes (McCauley, Pickles, et al. 2020). In short, there is a need for studies that broadly conceptualize positive adult outcomes for autistic individuals and those with related developmental conditions (Lounds Taylor 2017).

## 3 The Current Study

To understand associations between specific daily living skills in childhood and adolescence and specific adult outcomes, this study examined item set scores on the DLS domain of the Vineland Adaptive Behavior Scales (VABS; Sparrow et al. 1984, 2005) in a well‐characterized longitudinal cohort. By examining DLS at the level of item sets, this study provides insight into which DLS are most important to supporting adult outcomes. To understand adult outcomes, the current study examines subjective well‐being as a more holistic and inclusive indicator of adult life, alongside employment, living status, and social relationships. The aims and hypotheses were as follows:

1. Examine growth patterns in mean VABS DLS item set scores from ages 5, 9, 14, and 18. Given existing evidence that adaptive behavior growth varies with intellectual ability (Alvares et al. 2020; Smith et al. 2012), growth patterns were examined separately for individuals with IQ ≥ 70 and individuals with IQ < 70.
  It is hypothesized that growth patterns will vary meaningfully based on cognitive ability. Specifically, individuals with IQ ≥ 70 are expected to have steeper growth trajectories on all DLS item sets than individuals with IQ < 70.
2. Identify mean VABS DLS item set scores from ages 5, 9, 14, and 18 that—controlling for IQ, autism features, and other salient characteristics—predict adult outcomes (i.e., employment, living status, social relationships, and subjective well‐being) at approximately age 33 via multiple hierarchical and binary logistic regression.
  It is hypothesized that DLS item set scores from childhood will predict adult outcomes. However, given limited existing work in this area, there are no a priori hypotheses about which item set scores from childhood will predict which adult outcomes.

## 4 Method

### 4.1 Sample

Consecutive referrals (*n* = 213) under 37 months old at clinics in North Carolina and Chicago enrolled in a longitudinal study of autism. One hundred ninety‐two participants were referred for autism, and 21 participants were referred for non‐spectrum developmental delays. Forty children of similar characteristics from Michigan joined the study at age 9 and were subsequently seen at the same times, for a total of 253 participants. Detailed descriptions of this cohort can be found in prior work (Anderson et al. 2014; Lord et al. 2006).

The current analyses include data from 230 individuals from this cohort. To be included in this study, participants had to have completed the Vineland at least once between the ages of 5 and 18. Thirty percent of the sample was from a community of color (26% Black, 1.3% Hispanic, 1.3% Asian, 0.9% multiracial, and 0.5% American Indian) and 19% of the sample was female (Table 2). Participants with missing data were likelier to have enrolled in the Chicago clinic (*p* = 0.03, Table 2). There were no significant differences in race, caregiver education, diagnosis, gender, or urbanicity between participants included in these analyses and the full longitudinal cohort (all *p* < 0.05, Table 2). However, prior analyses of this cohort found participants lost to follow‐up are more likely to be people of color and to have a primary caregiver with less than a 4‐year college degree (see Clarke, Sterrett, et al. 2021; Pickles et al. 2020). Thus, all analyses included recruitment site, race, and caregiver education as covariates.

**TABLE 2:** Descriptive characteristics of the current subsample and total longitudinal cohort.

		Current subsample (*n* = 230)		Total cohort (*n* = 253)		
		Autism	DD^a^	Autism	DD^a^	
Race	White	126	34	135	40	*X* ^2^(1, 253) = 0.21, *p* = 0.64
	Person of color	53	13	60	12	
Ethnicity	Non‐Hispanic or Latino	176	47	188	54	
	Hispanic or Latino	2	1	5	3	
Sex	Male	158	28	170	33	*X* ^2^(1, 253) = 0.63, *p* = 0.42
	Female	23	21	26	24	
Caregiver education	< 4‐year degree	90	30	89	15	*X* ^2^(1, 253) = 2.54, *p* = 0.11
	≥ 4‐year degree	91	19	89	27	
Ability level	MA^b^	64	28	67	27	*X* ^2^(1, 228) = 0.08, *p* = 0.77
	LA^c^	111	18	115	20	
Urbanicity	Urban	98	29	108	36	*X* ^2^(1, 253) = 2.90, *p* = 0.08
	Rural	82	20	87	21	
Recruitment site	North Carolina	100	26	105	27	*X* ^2^(2, 253) = 6.96, *p* = 0.03
	Chicago	66	4	74	7	
	Michigan	15	19	17	23	

*Note:* Of the people of color in the current subsample, 60 identified as Black, 3 as Asian, 2 as multiracial, and 1 as American Indian. Chi‐square was not calculated for ethnicity due to insufficient *n*.

^a^ Non‐spectrum developmental disability.

^b^ More cognitively able.

^c^ Less cognitively able.

As in many longitudinal samples, most participants in the LSA sample did not provide data at every time point of the study. Currently, more than 30 years since the cohort's inception, about 157 participants and their families continue to provide data regularly. This is comparable to attrition rates in other existing longitudinal samples (Gustavson et al. 2012). Thus, the available *n* for adult outcome measures is smaller than the sample *n* and varies across measures. The available sample for each adult outcome measure is reported in Table 3.

**TABLE 3:** Correlations between adult outcome measures in the LSA sample.

Measure	*n*	1	2	3	4
Employment	152	—			
2 Living status	170	0.646***	—		
3 Social relationships	159	0.551**	0.396**	—	
4 Happiness factor	111	0.233*	0.344***	0.252**	—

*** *p* < 0.001.

** *p* < 0.01.

* *p* < 0.05.

### 4.2 Procedure

Research‐reliable clinicians administered the VABS, IQ testing, and other measures of interest during face‐to‐face visits at ages 5, 9, and 18. Age 5 visits were conducted in 1996, age 9 visits were conducted in 2000, and age 18 visits were conducted in 2009. The VABS was also completed via phone interview at approximately age 14. Adult outcome data was collected via mailed survey packets completed by caregivers and participants capable of self‐report at approximately age 33; this data was collected in 2023. Caregivers and participants over 18 who were their own legal guardians signed consent forms as required by the applicable institutional review board(s) prior to each visit. Ethical approval for this research was obtained from the Institutional Review Boards at the various study sites (UCLA, University of Michigan, University of Chicago, and University of North Carolina, Chapel Hill).

### 4.3 Measures

#### 4.3.1 Daily Living Skills

The Vineland Adaptive Behavior Scales (VABS; Sparrow et al. 2005) clinician interview was administered at nine time points: ages 2, 3, 5, 9, 14, 18, 21, 26, and 30. To assess whether DLS in childhood and adolescence could predict adult outcomes, the current study analyzed data from four time points: ages 5, 9, 14, and 18. Prior analyses in this cohort suggest VABS scores from early childhood had little predictive utility (Pickles et al. 2020); thus, VABS data from ages 2 and 3 were not included here. Participants completed the first edition of the VABS (Sparrow et al. 1984) from ages 2–9 and the second edition (Sparrow et al. 2005) from ages 14–18. For ease of interpretability, only DLS items that were equivalent across the first and second editions were included in the current analyses (Table 1).

The Vineland administration rules state that four consecutive items with a score of 2 (indicating the participant “Usually” completes the DLS in question independently) are considered basal, and four consecutive items with a score of 0 (indicating the participant “Never” completes the DLS in question independently) are considered ceiling. Participants' item set scores were calculated as the average of all items included within each set. Thus, the maximum possible score was 2 and the minimum possible score was 0 for each item set. Notably, item set scores were thus more restricted in variability than VABS raw scores or v‐scale scores. For analytic purposes, DLS items that were not administered but preceded a participant's basal items were assigned a score of 2, and items not administered that followed a participant's ceiling items were assigned a score of 0.

#### 4.3.2 IQ

The Mullen Scales of Early Learning (Mullen 1995) were administered at age 2. Later cognitive assessments were chosen from a standard hierarchy, which included the Weschler Intelligence Scale for Children (WISC; Wechsler 2003), Wechsler Abbreviated Scale of Intelligence (WASI; Wechsler 2011), Differential Abilities Scale (DAS; Elliott 2007), and Mullen based on participants' developmental abilities (Lord et al. 2006). Ratio IQs were calculated when raw scores fell outside deviation score ranges (see Anderson et al. 2014). Prior analyses of this cohort have distinguished participants who are “more cognitively able,” meaning their verbal IQ ≥ 70, and participants who are “less cognitively able,” meaning their verbal IQ is < 70 (McCauley, Elias, et al. 2020). Of the 230 participants included in the current study, 92 are more cognitively able (MA), and 129 are less cognitively able (LA). Nine participants in this subsample did not have IQ data at or after age 9.

#### 4.3.3 Autism Features

Autism Diagnostic Observation Schedule (ADOS‐2; Lord et al. 2012) calibrated severity scores (CSS) from age 9 were used to measure core autism features. ADOS CSS denotes an individual's autism features ranging from 10 (clearest case of autism with many features) to 1 (no evidence of autism).

Despite multiple assessments and a history of developmental delay, 49 individuals in this sample were not diagnosed with autism by our research team, blind to previous diagnoses, but instead received other diagnoses (including ADHD, Down's Syndrome, and non‐verbal learning disability). Prior analyses found participants with and without autism in this sample do not differ on a range of characteristics, including the likelihood of having social contacts, living independently, and working in adulthood (Clarke and Lord 2023; McCauley, Pickles, et al. 2020). Given considerable overlap in the challenges faced during adulthood for all individuals with developmental conditions (Lord et al. 2020), participants with non‐spectrum delays were retained in the current analyses.

#### 4.3.4 Adult Outcomes

The available sample size for each adult outcome measure and correlations between adult outcome measures are depicted in Table 3. Descriptive information on the adult outcomes of the current sample is included in Table 4.

**TABLE 4:** Adult outcome attainment by ability level and diagnostic status.

		MA		LA	
		Autism	DD	Autism	DD
Employment—vocational index score m (SD)		8.50 (2.69)	8.41 (2.85)	1.99 (1.78)	2.83 (0.69)
Living status	Living with support	29	8	74	12
	Living independently	27	11	1	0
Social relationships^a^	At least one social relationship	41	14	13	5
	No social relationships	12	4	59	5
Subjective well‐being—happiness factor score m (SD)		0.01 (0.81)	0.76 (0.60)	−0.20 (0.76)	0.64 (0.69)

^a^ For MA participants, a social relationship was defined as a mutual peer friendship. For LA participants, a social relationship was defined as a regular social contact who was not an immediate family member or paid staff person.

### 4.4 Employment

Employment data from demographic forms were coded using the Vocational Index (VDI) developed by Taylor and Seltzer (2012). Scores on the VDI range from 1 to 9, with 1 indicating no participation in employment or post‐secondary education activities, and 9 indicating participation in post‐secondary education or competitive employment for 10 h/week or more without supports. The VDI also includes scores for individuals in volunteer, sheltered workshop, and supported employment settings, making it an effective measure for understanding vocational outcomes among individuals of varying abilities (Taylor and Seltzer 2012). A modified version of the VDI that includes a score of 10 was used for the current analyses (see Clarke, Sterrett, et al. 2021). A score of 10 was defined as participation in competitive employment for 20 h/week or more without supports.

### 4.5 Living Status

Data from demographic forms was collapsed into a binary variable, with 1 indicating the participant currently lived independently (this included living with roommates or romantic partners) and 0 indicating the participant currently lived in the family home or a supported residential setting (e.g., group home, or a facility with designated support staff).

### 4.6 Social Relationships

The social experiences of MA and LA participants vary substantially; thus, different indicators of friendship were used for MA and LA participants. A positive friendship outcome was defined as having at least one mutual friendship for MA individuals. The Social Emotional Functioning Interview (SEF‐S & SEF‐I; Rutter 1988) was used to assess MA participants' friendships. Question 28 of the SEF, “Friends,” asks respondents, “Does [the participant] have any particular friends whom he/she sees? Who are they? Do these people ever come to his/her home or does he/she usually meet them at the club, center, etc.?” Data from SEF question 28 was collapsed into a binary variable, with 1 indicating at least one mutual friendship and 0 indicating no mutual friendships.

A positive friendship outcome for LA individuals was defined as having at least one regular social contact outside of family members and paid staff. In other words, a social contact was defined as a person who regularly spent time with the participant and was not either (1) an immediate relation or (2) being paid for providing a service to the participant. Information from demographic forms was collapsed into a binary variable, with 1 indicating at least one social relationship and 0 indicating no social relationships.

### 4.7 Subjective Well‐Being

Prior exploratory factor analysis in this sample (McCauley, Pickles, et al. 2020) identified a “happiness quotient” factor separately in both caregiver and self‐report data consisting of scores from the positive affect scale of the Positive and Negative Affect Scales (PANAS; Watson et al. 1988), the total score from the Scales of Psychological Well‐Being (WBQ; Ryff 1989), and the total score from the Quality of Life Questionnaire (QLQ; Schalock and Keith 1993). Scores on all three subjective well‐being measures were scaled and averaged to produce a “happiness quotient” composite score (McCauley, Pickles, et al. 2020). Happiness quotient composite scores ranged from −1 to 1, with higher scores indicating greater reported levels of subjective well‐being. Prior work in this cohort found that participation in employment activities (Clarke, Sterrett, et al. 2021) and relatively few symptoms of psychopathology (McCauley, Elias, et al. 2020) were associated with higher caregiver‐and self‐report happiness quotient scores. Only caregiver‐report scores were used in the current analyses, as this allowed for the inclusion of LA participants who could not self‐report on their internal states.

## 5 Analytic Plan

### 5.1 Aim 1

To characterize growth patterns in VABS DLS item set scores, visualizations were created using the ggplot2 package in R, version 4.3.0 (R Core Team 2023). Given the large number of item sets (15 in total; Table 1), the relatively small number of items within each set, and the a priori assumption that growth patterns would vary meaningfully based on cognitive ability (MA/LA), group‐based trajectory modeling was not conducted. Prior analyses of this sample have reported the results of group‐based trajectory models examining growth in DLS domain and subdomain‐level scores (Bal et al. 2015; Clarke, McCauley, et al. 2021).

### 5.2 Aim 2

#### 5.2.1 Power Analysis

An a priori power analysis was conducted using G*Power version 3.1.9.7 (Faul et al. 2007) to determine the minimum sample size required for Aim 2 regression analyses. Results indicated that to test all covariates and DLS item sets simultaneously, the required sample size was *n* = 160 to achieve 80% power for detecting a medium effect at a significance criterion of *α* = 0.05. Given the limited sample size for each adult outcome measure (See Table 3), the current study was underpowered to test all variables in a single block. Thus, as described below, regression analyses were conducted separately for covariate and DLS item sets. Additionally, the significance level for regression analyses was set at *p* ≤ 0.01.

#### 5.2.2 Regression Analyses

Regression analyses were used to examine whether Vineland DLS item set scores at ages 5, 9, 14, and 18 predicted employment, the likelihood of living independently, the presence of friendships, and well‐being at age 33. Multiple hierarchical regression was used for continuous outcome variables (“happiness quotient” and VDI scores). For categorical outcome variables (residential status, friendships), binary logistic regression was used. All regression analyses were conducted via SPSS 28.

First, to retain power while testing covariates, separate regressions for salient demographic (i.e., race, diagnosis, caregiver education, recruitment site) and phenotypic characteristics (i.e., IQ at 9, ADOS CSS score at 9) were run for each adult outcome. Phenotypic data from age 9 was used in the current analyses because evidence suggests IQ is often quite stable after mid‐childhood (Anderson et al. 2014) and core autism features peak in mid‐childhood (Masjedi et al. 2024; Seltzer et al. 2004). Variables identified as significant predictors during this step were included as covariates in VABS DLS item set regression models. When VABS DLS item sets that predicted attainment of a given adult outcome for the total sample were identified, additional regression analyses separated by ability level (MA/LA) were also conducted. Given the number of tests required, the significance level was set at *p* ≤ 0.01.

## 6 Results

### 6.1 Aim 1

**FIGURE 1:**
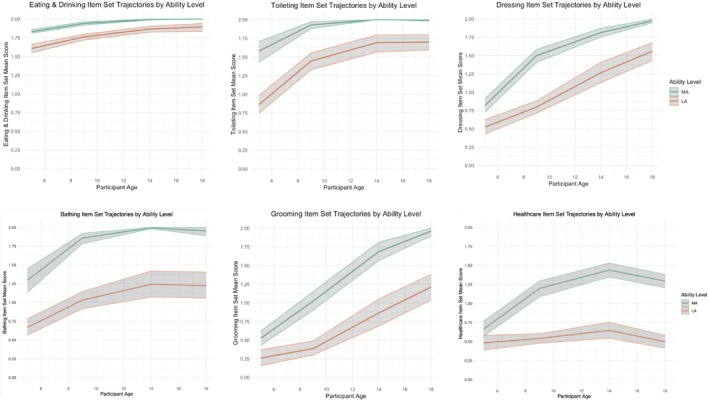
Patterns of growth in personal subdomain mean item set scores from ages 5–18.

**FIGURE 2:**
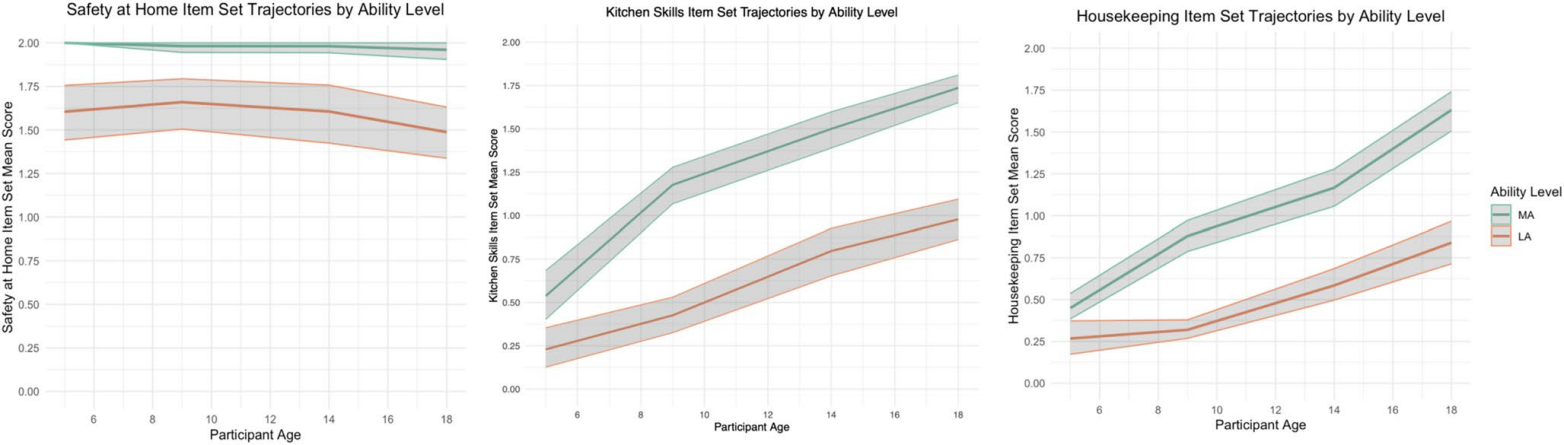
Patterns of growth in domestic subdomain mean item set scores from ages 5–18.

**FIGURE 3:**
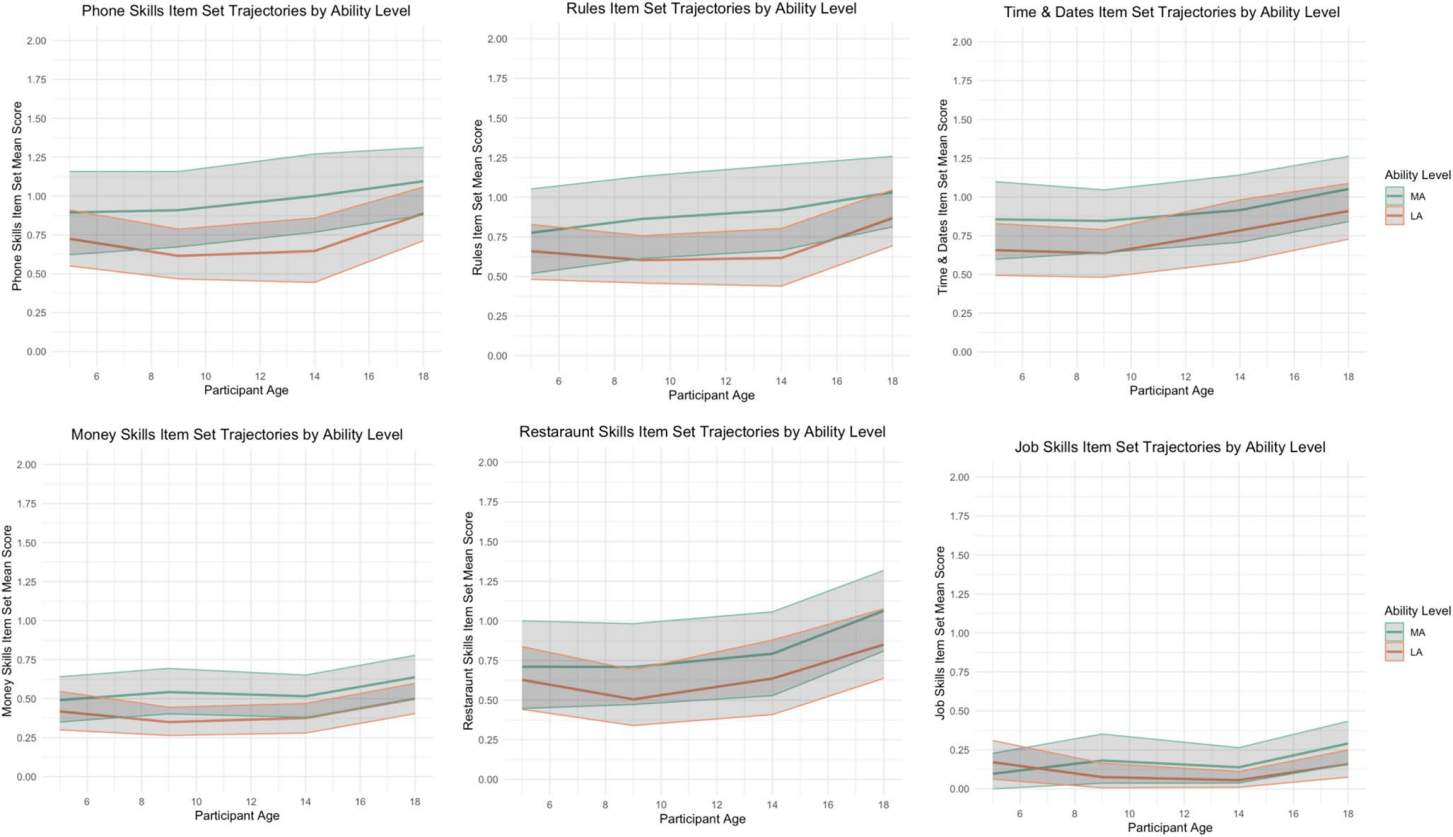
Trajectories of community subdomain item set scores from ages 5–18.

Patterns of growth in mean scores for DLS item sets from ages 5–18 are displayed in Figure 3. Data from all 230 participants was used to create the visuals in Figure 1; 179 participants had VABS data at two or more of the time points of interest (i.e., ages 5, 9, 14, and 18). MA participants had higher mean scores than LA participants for item sets in the personal (Figure 1) and domestic (Figure 2) subdomains. Excluding the health item set, MA participants reached ceiling (i.e., the maximum score) on all personal subdomain item sets in adolescence. Again, excluding the health item set, LA participants' scores on the personal subdomain item sets consistently improved with age, despite not reaching ceiling (Figure 1). In contrast, by age 18, the majority of both MA and LA participants were not independently completing DLS related to two item sets from the domestic subdomain: cooking and household chores (Figure 2).

Mean scores for item sets in the community subdomain were also quite low regardless of cognitive ability (Figure 3). By age 18, the highest mean scores for item sets in the community subdomain were slightly above 1, indicating participants “sometimes” completed these DLS independently. For the money and pre‐job skills item sets, mean scores at age 18 were lower than 1 (Figure 3). For both MA and LA participants, community DLS were an area of pronounced difficulty throughout childhood and adolescence.

### 6.2 Aim 2

Regression analyses for salient demographic (i.e., race, diagnosis, caregiver education, recruitment site) and phenotypic (i.e., IQ at 9, ADOS CSS score at 9) covariates are reported first, followed by analyses for each adult outcome of interest for the total sample. Finally, regression analyses separated by ability level (MA/LA) are reported. Given the number of tests required, the significance level was set at *p* ≤ 0.01.

#### 6.2.1 Covariates

IQ at age 9 was a significant predictor of VDI scores (*p* < 0.001), social relationships (*p* < 0.001), and residential status (*p* < 0.001). Additionally, diagnostic status (autism/non‐spectrum developmental delay) was a significant predictor of happiness quotient scores (*p* < 0.001). Race, caregiver education, recruitment site, and ADOS CSS at 9 did not significantly predict any adult outcomes (all *p* > 0.05). Thus, IQ at age 9 was included as a covariate in the first block of all VDI, friendship, and living status outcome regression models (Table S1), and diagnostic status was included in the first block of all happiness quotient score models (Table S2).

#### 6.2.2 Vocational Index Scores

Controlling for IQ at age 9, bathing (*p* = 0.003) and health (*p* = 0.007) mean item set scores from age 5 predicted VDI scores at age 33. From age 9, housekeeping (*p* = 0.006) mean item set scores predicted VDI scores. No DLS mean item set scores from age 14 predicted VDI scores. At age 18, pre‐job skills (*p* = 0.001) predicted VDI scores (Table S1). Given the significant findings for the total sample, groups were then considered separately by ability level.

Amongst LA participants, toileting (β = 0.305, *t*(83) = 3.23, *p* = 0.002), bathing (*p* < 0.001), and health (β = 0.262, *t*(83) = 2.81, *p* = 0.006) mean item set scores from age 5 were predictive of adult VDI scores. Grooming (β = 0.295, *t*(94) = 2.75, *p* = 0.007), phone skills (β = 0.239, *t*(94) = 2.64, *p* = 0.010), money skills (β = 0.289, *t*(94) = 3.26, *p* = 0.002), and Pre‐job skills (β = 0.422, *t*(94) = 5.12, *p* < 0.001) mean item set scores at age 9 were predictive of higher VDI scores at age 33. No DLS mean item set scores from ages 14 or 18 significantly predicted VDI scores at age 33 for LA participants. For MA participants, no mean item set scores from ages 5, 14, or 18 predicted VDI scores. Pre‐job skills (β = −0.363, *t*(52) = −2.86, *p* = 0.006) mean item set scores from age 9 significantly predicted higher VDI scores at age 33 for MA participants.

#### 6.2.3 Happiness Quotient Scores

Controlling for diagnostic status, no Vineland DLS item set mean scores from age 5 predicted happiness quotient scores. From age 9, bathing (*p* = 0.005) mean item set scores predicted happiness quotient scores. From age 14, bathing (*p* = 0.003) and health (*p* = 0.004) significantly predicted happiness quotient scores. Finally, housekeeping (*p* = 0.002) and pre‐job skills (*p* = 0.004) mean item set scores from age 18 significantly predicted happiness quotient scores (Table S2). Given the significant findings for the total sample, groups were then considered separately by ability level.

Amongst LA participants, bathing (β = 0.265, *t*(94) = 2.73, *p* = 0.008) mean item set scores at age 9 significantly predicted happiness quotient scores. Bathing (β = 0.341, *t*(63) = 2.99, *p* = 0.004) mean item set scores remained a significant predictor of happiness quotient scores for LA participants at age 14. Health (β = 0.398, *t*(63) = 3.59, *p* < 0.001) mean item set scores at age 14 were also a significant predictor of happiness quotient scores. There were no mean item set scores from age 18 that significantly predicted happiness quotient scores for LA individuals. For MA participants, no item set mean scores prior to age 18 were predictive of happiness quotient scores. At age 18, eating out skills (β = 0.323, *t*(59) = 2.78, *p* = 0.007) and pre‐job skills (β = 0.339, *t*(59) = 2.93, *p* = 0.005) mean item set scores significantly predicted happiness quotient scores for MA individuals.

#### 6.2.4 Friendships and Residential Status

Controlling for verbal IQ at age 9, no Vineland DLS item set mean scores from ages 5, 9, 14, or 18 predicted the likelihood of having friends or living independently. Thus, regression analyses by ability level were not conducted.

## 7 Discussion

With intervention and support, the DLS of autistic individuals and those with related developmental conditions can improve, and DLS interventions implemented in childhood and adolescence could provide foundational skills to support later adult success (Bennett and Dukes 2013; Duncan et al. 2018, 2023). But there is limited understanding of the specific DLS that are most important to supporting adult outcomes for those with developmental conditions. This study strove to address this gap by characterizing growth patterns in DLS item sets from mid‐childhood through adolescence and pinpointing specific daily living skills from ages 5, 9, 14, and 18 that predicted positive adult outcomes.

All participants experienced growth in their personal and domestic item set scores, and MA participants had higher mean scores than LA participants at all‐time points for these two subdomains (Figures 1 and 2). However, regardless of ability level, all participants had very low item set scores within the community subdomain, with limited growth over time (Figure 3). Daily living skills related to navigating “the real world”—such as answering a phone, making small purchases, and ordering in a restaurant—may be an area of relative weakness for many autistic individuals. Given that four of the six item sets from the community subdomain emerged as significant predictors of employment and/or well‐being in Aim 2 analyses, the limited growth in community DLS observed here is concerning. Notably, for community item set scores from age 18, there may be a conflation of predictor and outcome variables—one higher‐level item specifically asks about the ability to maintain employment, for example (Table 1).

For the sample as a whole, DLS mean item set scores from ages 5, 9, and 18 predicted employment at approximately age 33, and scores from ages 9,14, and 18 predicted well‐being. No DLS item sets were predictive of friendships and residential status. For LA participants, item sets from the personal subdomain had the most predictive utility. In contrast, item sets from the community subdomain had the most predictive utility for MA participants.

### 7.1 Growth in DLS Item Set Scores

Both LA and MA participants experienced linear growth in most personal and domestic item sets from ages 5–18. The health item set in the personal subdomain was a notable exception, with participants' scores decreasing from ages 14–18 (Figure 1). This item set assesses basic skills related to maintaining the personal health of oneself and others (i.e., blowing one's nose using a tissue; Table 1), so it is unclear why health item set scores declined during adolescence. One possibility is that the onset of puberty and subsequent increased and novel expectations regarding personal hygiene could result in declining maintenance of other hygiene tasks (Hamdan 2022). Strikingly, item sets from the community subdomain had the lowest mean scores in this sample, with the least growth over time (Figure 3). MA participants' mean item set scores from the community subdomain were not markedly higher than those of LA participants—this was the only subdomain in which this was the case.

### 7.2 Predictive Utility of DLS Item Set Scores

Only employment and subjective well‐being outcomes were predicted by DLS mean item set scores in the regression analyses. Two item sets from the personal subdomain—bathing and health—emerged as significant predictors of employment and subjective well‐being. These associations appeared to be driven primarily by LA participants. Interventions targeting skills related to personal hygiene (e.g., showering independently) and health (e.g., covering one's nose when coughing or sneezing) may promote quality of life and improve the likelihood of obtaining employment for autistic adults with co‐occurring intellectual disability. Given the diverse characteristics of MA and LA participants in this sample, it is logical that different types of DLS would be integral to adult success for these individuals. In contrast to LA participants, adult employment and well‐being outcomes for MA participants were associated with item sets from the community subdomain—eating out skills and pre‐job skills.

On average, participants with developmental conditions other than autism in the current sample had higher caregiver‐reported subjective well‐being scores than autistic participants. Prior work has found that caregivers of autistic children report lower well‐being than caregivers of children with Down's syndrome and Fragile X syndrome (Abbeduto et al. 2004). Though caregivers in the current study were asked to report on the well‐being of their adult family member, it is possible that their perceptions of their own well‐being may have influenced these scores. Importantly, this was the only significant difference found between diagnostic groups. Prior work has also found that there are more similarities than differences in the adult experiences of autistic individuals and individuals with other developmental conditions (Agarwal et al. 2022; Lord et al. 2020). Regardless of the specific diagnostic label, individuals with developmental conditions are more likely to experience challenges in adult life than their peers in the general population.

Notably, the life skills that predicted adult outcomes in the current sample may not in and of themselves increase the likelihood of attaining adult outcomes. In other words, in isolation, teaching an autistic adolescent how to shower independently is unlikely to ensure their well‐being in adulthood. Instead, perhaps the DLS identified as significant predictors in this study reflect profiles of individual strengths associated with later adult success. Another likely alternative is that growing up in a context where opportunities to develop such skills are regularly provided is a promotive factor that increases outcome attainment (see *The Role of Opportunity*, below). Additional research is needed to tease apart why the DLS identified here are associated with adult outcomes for autistic individuals.

No DLS item sets predicted adult friendships or living status. The use of dummy variables to measure these outcomes may have contributed to the null findings. Binary indicators do not allow for the same degree of nuance as continuous variables, such as those used to measure well‐being and employment outcomes. Categorical variables measuring friendship and living status were investigated, but the bimodal outcome distributions seen in this sample limited the utility of this approach. e.g., most participants either lived in the family home or a group home (i.e., a fully supported residential environment) or independently—few individuals lived with part‐time support or some other intermediary arrangement.

### 7.3 Clinical Implications

Though challenges with DLS are frequently present in autistic individuals, they are not a diagnostic criterion of the condition and have been infrequently considered as a primary outcome in intervention trials. In other words, far more is known about how DLS challenges manifest in autism than is known about how to improve DLS in autism (Clarke et al. 2025; Duncan et al. 2018). Two notable exceptions to this are the *Building Confidence* and *Surviving and Thriving in the Real World* (STRW) intervention programs, which have both demonstrated significant improvements in DLS in autistic children and adolescents (Drahota et al. 2011; Duncan et al. 2023). However, both programs are designed for autistic youth with average or better IQ. Outside of single‐case designs, little research has examined how to systematically improve DLS in autistic individuals with co‐occurring ID (Burns et al. 2019). It is also unclear whether there are impacts on the adult outcomes and DLS of autistic youth who participate in such interventions; long‐term follow‐up studies are needed to determine this.

Frequently, behavioral interventions are limited to a specific context, such as the home, classroom, or clinic. None of these contexts may be conducive to learning community DLS, which these results suggest is an area of weakness for many individuals with developmental conditions. Providing opportunities for individuals to learn and practice DLS in public settings may promote positive adult outcomes. Conducting an educational or therapy session in a grocery store or at a café, for example, may provide far more opportunity for a client to learn and practice community daily living skills than conducting a similar session in school or the clinic (Glover et al. 2023). Similarly, telehealth approaches may facilitate opportunities to practice community DLS, as clinicians could provide real‐time feedback to clients navigating a grocery store, bus stop, or other community settings from the comfort of their office (Clarke et al. 2025).

In addition to targeting community daily living skills, ensuring individuals are proficient in skills related to personal hygiene and health may improve adult outcomes for autistic adults with co‐occurring intellectual disability. Given that these daily living skills can be taught and practiced at home with relative ease, clinicians could support caregivers in assisting their autistic family member to practice hygiene and health skills by building such practice into preexisting daily routines (i.e., showering before bedtime).

### 7.4 The Role of Opportunity

Individuals with developmental conditions may need more opportunities, sometimes including concrete instruction, to develop competencies in life skills compared to the general population. Still, it would be difficult for anyone to learn a new skill without opportunities to learn and practice. Recent evidence suggests that for autistic adults of all abilities, lack of opportunity to practice is a common barrier to improving DLS (Teh et al. 2024).

Some individuals with developmental conditions may be subject to higher expectations for their DLS competency than others, resulting in more opportunities to learn and practice. These higher expectations could arise from a confluence of factors, including personal traits, characteristics of the family, school, and/or work environment, and access to services and supports (unfortunately, information on the latter was not available for the current analyses; see *Limitations* below). For example, an autistic child with an average IQ who is placed in an inclusive classroom may have higher expectations placed upon them by teachers and peers than an autistic child with co‐occurring ID placed in a segregated school setting. Similarly, an autistic individual with co‐occurring ID living in a family that expects self‐sufficiency may acquire competency in DLS at a faster pace than an autistic individual with average cognitive abilities whose family does not have similar expectations. In both cases, expectations inform opportunities for DLS practice.

## 8 Limitations

This sample comprises a unique group of adults with autism and related developmental conditions. Participants' families sought help early in childhood during the early 1990s, an era in which autism awareness was much less widespread than it is today. Thus, these participants may be different from both younger individuals and same‐aged individuals diagnosed later in development (Lord et al. 2022). Attrition has reduced the number of participants in this cohort, particularly those who are Black and those with lower caregiver education. Additionally, we have few female participants, which constrained the ability to test for gender differences in these analyses. The current sample may have been underpowered for the Aim 2 regression analyses. With this in mind, the results of Aim 2 should be considered exploratory in nature. Future work should attempt to replicate Aim 2 results in appropriately powered independent samples of adults with developmental conditions.

Unfortunately, data on the amount and types of services participants received in childhood were unavailable for the current project. It is reasonable to assume that services receipt could meaningfully inform opportunities to learn and practice DLS. Thus, the amount and types of services participants in the current sample received are important unmeasured variables to consider in the interpretation of these findings. Additionally, subjective well‐being was only measured via caregiver report in the current analyses. This allowed for the inclusion of participants who could not report on their own internal states in the well‐being analyses, which is a strength of this work. However, prior literature suggests discrepancies between caregiver and self‐report quality of life for autistic adults (McCauley et al. 2025; Sandercock et al. 2020).

Although widely used, the Vineland Adaptive Behavior Scales is not a perfect measure. Recent evidence in the general population suggests female children score significantly higher on the VABS than similarly aged male children (Nishimura et al. 2022), but the Vineland is not gender normed. There is also evidence of cross‐cultural differences in Vineland scores even across high‐income, Western nations (Fombonne and Achard 1993; Touil et al. 2021). Including data from multiple versions of the Vineland is also a limitation of this study. The current analyses used data from the first and second editions of the VABS and did not include data from the third edition of the VABS.

The number of items within each item set on the Vineland varies. This is particularly true for the purposes of this study because only items from the first and second editions of the Vineland that completely overlapped were retained for analyses. The largest item sets here, *Eating and Drinking* and *Housekeeping*, include nine items each. In contrast, the smallest item sets here, *Safety at Home* and *Eating Out Skills*, contain only one item each. Thus, the variance within item sets and the likelihood that item set scores represent “true” domain scores may vary considerably. This is a weakness of the current study, reflecting the fact that VABS item set scores are not normed to be individually analyzed and interpreted. The results of this project should be considered with this in mind.

## 9 Future Directions

For all individuals with developmental conditions, including autism, increasing opportunities to learn and practice specific daily living skills may result in improved DLS (Teh et al. 2024). Caregivers play an integral role in determining how many and what kinds of opportunities autistic individuals have to practice DLS in the home environment. One possible avenue for future work in this area would be to characterize the quantity and quality of supports caregivers provide to facilitate DLS acquisition. Similar work investigating supports provided by teachers and 1:1 support staff in the school environment could also enhance understanding of how to promote opportunities for autistic individuals to learn and practice DLS.

This study only analyzed data from the daily living skills domain of the Vineland Adaptive Behavior scales. The predictive utility of item sets from other domains of the VABS should be explored in future work. Additional work is needed to understand how differences between Vineland editions impact assessment scoring and interpretation. There is some evidence that the Adaptive Behavior Assessment Scale (ABAS; Harrison and Oakland 2015) is a more valid and reliable measure of adaptive behavior for autistic adults. However, little work to date has explored how ABAS scores relate to concurrent or later outcomes for autistic adults. There are also only a handful of studies directly comparing the convergent reliability of the VABS and the ABAS (Tamm et al. 2022), and almost no work has detailed item‐level analyses of the ABAS. These all represent important areas for future inquiry.

## 10 Conclusion

Decades of research have carefully documented DLS challenges in autism and related developmental conditions, but this work has provided little insight into how specific DLS change over time or relate to adult outcomes. The current study addressed this gap by characterizing growth in DLS mean item scores from ages 5–18 and by identifying DLS item sets from ages 5, 9, 14, and 18 that predicted adult outcomes. Participants experienced limited growth in community DLS item set scores from ages 5–18, regardless of cognitive ability. DLS item set scores predicted adult employment and well‐being outcomes. For LA participants, DLS from the personal subdomain (i.e., bathing, health) had the most predictive utility. In contrast, for MA participants, DLS from the community subdomain (i.e., eating out skills, pre‐job skills) had the most predictive utility. These findings shed light on which DLS are most important to supporting adult success and could be used to inform meaningful treatment and educational goals for individuals with autism and related developmental conditions.

## Conflicts of Interest

C.L. acknowledges the receipt of royalties from the sale of the Autism Diagnostic Observation Schedule (ADOS) and the Autism Diagnostic Interview‐Revised (ADI‐R). Royalties generated from this study were donated to a not‐for‐profit agency, Have Dreams. E.B.C. has no potential conflicts to declare.

## Supporting information


**Table S1.** Regression models predicting adult employment from mean DLS item set scores at ages 5, 9, 14, and 18.
**Table S2.** Regression models predicting adult well‐being from mean DLS item set scores at ages 5, 9, 14, and 18.

## Data Availability

The data that support the findings of this study are available from the corresponding author upon reasonable request.

## Endnotes

^1^
In accordance with mixed preferences, and in recognition that the sample described in this study includes both individuals who view autism as a component of their identity as well as individuals who are profoundly impacted by their autism, we refer to this population interchangeably as “autistic individuals” and “individuals with autism” throughout.
