# Supplementary material for: Identifying Daily Living Skills From Childhood and Adolescence Predictive of Adult Outcomes in a Longitudinal Study of Autism and Related Developmental Conditions
Source: Autism Res. 2025 May 30;18(7):1474–88. doi: 10.1002/aur.70056 (PMC12279000; doi:10.1002/aur.70056)
Supplement: Supplementary file 1 — Table S1. Regression models predicting adult employment from mean DLS item set scores at ages 5, 9, 14, and 18. Table S2. Regression models predicting adult well‐being from mean DLS item set scores at ages 5, 9, 14, and 18. [file AUR-18-1474-s001.doc]

**Table S1.** Regression Models Predicting Adult Employment from Mean DLS Item Set Scores at Ages 5, 9, 14, and 18.

|  | Age 5  *n* = 124 | | | | Age 9  *n* = 152 | | | | Age 14  *n* = 120 | | | | Age 18  *n* = 143 | | | |
| --- | --- | --- | --- | --- | --- | --- | --- | --- | --- | --- | --- | --- | --- | --- | --- | --- |
| Step 1: covariates | | | | | | | | | | | | | | | | |
|  | *B* | SE*_B_* | 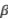 | 95% CI | *B* | SE*_B_* | 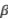 | 95% CI | *B* | SE*_B_* | 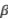 | 95% CI | *B* | SE*_B_* | 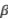 | 95% CI |
| Intercept | -.22 | .31 |  | [-.85, .41] | -.14 | .33 |  | [-.80, .51] | .66 | .43 |  | [-.19, 1.52] | .04 | .38 |  | [-.71, .80] |
| IQ | .08 | .005 | .83 | [.07, .09] | .08 | .005 | .80 | [.07, .09] | .07 | .006 | .75 | [.06, .08] | .07 | .005 | .77 | [.06, .09] |
| Step 2: mean item set scores | | | | | | | | | | | | | | | | |
| Domestic Subdomain | | | | | | | | | | | | | | | | |
| Safety at home | .24 | .30 | .04 | [-.35, .85] | -.22 | .34 | -.03 | [-.90, .46] | -.50 | .46 | -.07 | [-1.41, .41] | -.37 | .41 | -.05 | [-1.18, .44] |
| Kitchen Chores | .90* | .35 | .12 | [.20, 1.59] | .48 | .44 | .07 | [-.38, 1.36] | .06 | .52 | .01 | [-.96, 1.09] | 1.05* | .49 | .15 | [.08, 2.02] |
| Housekeeping | .83 | .43 | .09 | [-.02, 1.70] | **1.74**** | .62 | .18 | [.51, 2.97] | .35 | .57 | .04 | [-.79, 1.49] | .81* | .39 | .13 | [.02, 1.60] |
| Personal Subdomain | | | | | | | | | | | | | | | | |
| Eating & Drinking | .97 | .91 | .06 | [-.83, 2.78] | .22 | 1.27 | .01 | [-2.30, 2.74] | -.05 | 1.92 | -.002 | [-3.86, .3.75] | .15 | 1.15 | .007 | [-2.13, 2.44] |
| Toileting | .80* | .34 | .13 | [.11, 1.48] | -.31 | .43 | -.04 | [-1.16, .54] | -.32 | .70 | -.03 | [-1.71, 1.06] | .13 | .57 | .01 | [-.99, 1.27] |
| Dressing | .89* | .43 | .10 | [.03, 1.76] | .42 | .52 | .05 | [-.60, 1.45] | -.07 | .54 | -.01 | [-1.15, 1.01] | .16 | .52 | .01 | [-.87, 1.19] |
| Bathing | **1.13**** | .37 | .17 | [.39, 1.87] | .22 | .41 | .03 | [-.60, 1.05] | .38 | .50 | .06 | [-.61, 1.38] | .35 | .35 | .06 | [-.34, 1.04] |
| Grooming | .80 | .41 | .10 | [-.01, 1.62] | .65 | .40 | .09 | [-.13, 1.44] | .09 | .40 | .01 | [-.70, .89] | .19 | .36 | .03 | [-.52, .91] |
| Health | **1.21**** | .43 | .13 | [.34, 2.08] | .95 | .56 | .11 | [-.16, 2.07] | .85 | .62 | .12 | [-.38, 2.10] | .98 | .55 | .13 | [-.11, 2.08] |
| Community Subdomain | | | | | | | | | | | | | | | | |
|  | Age 5 | | | | Age 9 | | | | Age 14 | | | | Age 18 | | | |
|  | *B* | SE*_B_* | 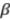 | 95% CI | *B* | SE*_B_* | 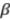 | 95% CI | *B* | SE*_B_* | 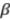 | 95% CI | *B* | SE*_B_* | 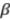 | 95% CI |
| Telephone Skills | .48* | .23 | .10 | [.02, .95] | .58* | .25 | .16 | [.08, 1.09] | .61 | .37 | .16 | [-.12, 1.34] | .12 | .41 | .02 | [-.69, .94] |
| Rules, rights, & safety | .57* | .24 | .11 | [.08, 1.05] | **.98***** | .25 | .30 | [.48, 1.48] | .58 | .34 | .15 | [-.10, 1.27] | .25 | .41 | .04 | [-.56, 1.07] |
| Time & Dates | .81* | .40 | .10 | [.006, 1.61] | .84* | .38 | .16 | [.09, 1.60] | .09 | .40 | .01 | [-.70, .89] | .17 | .38 | .03 | [-.59, .94] |
| Money skills | .40 | .27 | .07 | [-.14, .95] | .77 | .39 | .12 | [-.005, 1.55] | .13 | .51 | .02 | [-.88, 1.15] | **1.70**** | .61 | .22 | [.48, 2.92] |
| Eating Out skills | .54 | .35 | .07 | [-.16, 1.26] | **.74**** | .27 | .18 | [.20, 1.28] | .05 | .34 | .01 | [-.62, .73] | .01 | .29 | .002 | [-.57, .59] |
| Pre-job skills | .26 | .23 | .05 | [-.20, .72] | .31 | .31 | .04 | [-.31, .93] | -.17 | .47 | -.02 | [-1.11, .76] | **1.76***** | .51 | .24 | [.73, 2.78] |

Note. Given the number of tests required, the significance level for regression analyses was set at p ≤ .01.

* p < .05

** p < .01

*** p < .001

**Table S2.** Regression Models Predicting Adult Well-Being from Mean DLS Item Set Scores at Ages 5, 9, 14, and 18.

|  | Age 5  *n* = 124 | | | | Age 9  *n* = 152 | | | | Age 14  *n* = 120 | | | | Age 18  *n* = 143 | | | |
| --- | --- | --- | --- | --- | --- | --- | --- | --- | --- | --- | --- | --- | --- | --- | --- | --- |
| Step 1: covariates | | | | | | | | | | | | | | | | |
|  | *B* | SE*_B_* | 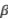 | 95% CI | *B* | SE*_B_* | 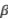 | 95% CI | *B* | SE*_B_* | 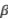 | 95% CI | *B* | SE*_B_* | 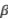 | 95% CI |
| Intercept | .55 | .15 |  | [-.85, .41] | .61 | .16 |  | [-.80, .51] | .52 | .15 |  | [.21, .83] | .56 | .15 |  | [.26, .86] |
| Diagnosis | -.43 | .16 | -.22 | [.07, .09] | -.58 | .17 | -.25 | [.07, .09] | -.56 | .17 | -.28 | [-.90, -.21] | -.59 | .16 | -.28 | [-.92, -.26] |
| Step 2: mean item set scores | | | | | | | | | | | | | | | | |
| Domestic Subdomain | | | | | | | | | | | | | | | | |
| Safety at home | .006 | .10 | .005 | [-.20, .21] | .18 | .10 | .13 | [-.02, .40] | .27* | .11 | .19 | [-.03, .51] | .12 | .11 | .09 | [-.09, .35] |
| Kitchen Chores | .06 | .12 | .04 | [-.18, .32] | .27* | .10 | .19 | [.05, .48] | .19 | .11 | .15 | [-.02, .42] | .22 | .11 | .15 | [-.002, .45] |
| Housekeeping | .01 | .16 | .01 | [-.30, .33] | .23 | .15 | .12 | [-.07, .55] | .24 | .13 | .16 | [-.02, .52] | **.32***** | .09 | .26 | [.13, .51] |
| Personal Subdomain | | | | | | | | | | | | | | | | |
| Eating & Drinking | .57* | .28 | .17 | [-.001, 1.14] | .55 | .35 | .12 | [-.15, 1.26] | .51 | .47 | .09 | [-.41, 1.44] | .09 | .35 | .02 | [-.60, .78] |
| Toileting | .16 | .10 | .13 | [-.05, .37] | .20 | .12 | .13 | [-.03, .45] | .24 | .17 | .12 | [-.09, .59] | .07 | .16 | .03 | [-.25, .39] |
| Dressing | .18 | .15 | .10 | [-.12, .49] | .29* | .12 | .18 | [.04, .53] | .04 | .12 | .03 | [-.20, .30] | .14 | .14 | .08 | [-.13, .42] |
| Bathing | .16 | .12 | .12 | [-.07, .40] | **.28**** | .10 | .22 | [.08, .47] | **.31**** | .10 | .26 | [-.10, .52] | .17 | .09 | .15 | [-.004, .35] |
| Grooming | .11 | .15 | .07 | [-.18, .41] | .26* | .11 | .18 | [.04, .48] | .09 | .09 | .08 | [-.09, .27] | .18* | .09 | .16 | [.001, .36] |
| Health | .002 | .16 | .001 | [-.32, .33] | .21 | .13 | .12 | [-.06, .48] | **.35**** | .12 | .25 | [.11, .59] | .29* | .11 | .20 | [.06, .53] |
| Community Subdomain | | | | | | | | | | | | | | | | |
|  | Age 5 | | | | Age 9 | | | | Age 14 | | | | Age 18 | | | |
|  | *B* | SE*_B_* | 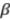 | 95% CI | *B* | SE*_B_* | 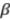 | 95% CI | *B* | SE*_B_* | 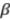 | 95% CI | *B* | SE*_B_* | 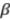 | 95% CI |
| Telephone Skills | .04 | .08 | .05 | [-.12, .22] | .12* | .06 | .16 | [.007, .24] | .15* | .06 | .22 | [.03, .28] | .14 | .09 | .13 | [-.03, .32] |
| Rules, Rights, & Safety | .04 | .09 | .04 | [-.13, .22] | **.**08 | .05 | .12 | [-.02, .19] | .14* | .06 | .19 | [.01, .27] | .19* | .09 | .16 | [.004, .38] |
| Time & Dates | .07 | .15 | .04 | [-.22, .36] | .16* | .08 | .15 | [.002, .33] | .16* | .08 | .16 | [-.007, .32] | .11 | .09 | .10 | [-.06, .29] |
| Money Skills | -.04 | .10 | -.03 | [-.24, .16] | .09 | .10 | .06 | [-.11, .30] | .15 | .11 | .12 | [-.06, .37] | .30* | .12 | .18 | [.05, .55] |
| Restaurant Skills | -.02 | .13 | -.01 | [-.28, .23] | .09 | .06 | .11 | [-.03, .23] | **.20**** | .07 | .25 | [.06, .34] | .05 | .07 | .05 | [-.09, .19] |
| Pre-job Skills | -.06 | .08 | -.06 | [-.22, .10] | -.01 | .10 | -.008 | [-.22, .20] | .002 | .13 | .002 | [-.26, .26] | **.34**** | .12 | .22 | [.10, .58] |

Note. Given the number of tests required, the significance level for regression analyses was set at p ≤ .01.

* p < .05

** p < .01

*** p < .001
